# Different approaches to Imaging Mass Cytometry data analysis

**DOI:** 10.1093/bioadv/vbad046

**Published:** 2023-04-03

**Authors:** Vladan Milosevic

**Affiliations:** Department of Clinical Medicine, Centre for Cancer Biomarkers CCBIO, University of Bergen, Bergen 5020, Norway

## Abstract

Imaging Mass Cytometry (IMC) is a novel, high multiplexing imaging platform capable of simultaneously detecting and visualizing up to 40 different protein targets. It is a strong asset available for in-depth study of histology and pathophysiology of the tissues. Bearing in mind the robustness of this technique and the high spatial context of the data it gives, it is especially valuable in studying the biology of cancer and tumor microenvironment. IMC-derived data are not classical micrographic images, and due to the characteristics of the data obtained using IMC, the image analysis approach, in this case, can diverge to a certain degree from the classical image analysis pipelines. As the number of publications based on the IMC is on the rise, this trend is also followed by an increase in the number of available methodologies designated solely to IMC-derived data analysis. This review has for an aim to give a systematic synopsis of all the available classical image analysis tools and pipelines useful to be employed for IMC data analysis and give an overview of tools intentionally developed solely for this purpose, easing the choice to researchers of selecting the most suitable methodologies for a specific type of analysis desired.

## 1 Introduction

Imaging Mass Cytometry (IMC) has arisen as a powerful tool for studying complex tissue morphology. With the possibility of detection and visualization of more than 40 different markers simultaneously, it is a strong asset that can help in understanding the biology of the tissues and is an advantageous tool in studying pathological processes (Chang *et al.*, 2016; Herdlevær *et al.*, 2022; Krop *et al.*, 2022; Kuett *et al.*, 2022; Rendeiro *et al.*, 2021; Wu *et al.*, 2021).

Along with the evolution of high multiplex imaging techniques, the development of different pipelines designed to analyze this particular type of data was also advancing. Taking into consideration the number of tools available for data analysis and their continuously increasing number, one might become lost in the field of overflowing options and face difficulties in deciding which of the available tools would be the best to use depending on data characteristics and scientific questions one wants to answer.

This review aims to give a systematic synopsis of all the available classical image analysis tools and pipelines useful to be employed for IMC data analysis and give an overview of tools intentionally developed solely for this purpose. In addition, the author will try to provide an overview of the strengths and limitations of each of these tools, bring all available options closer to scientists working with IMC data, and ease the choice of selecting the most suitable approach for a specific type of analysis desired.

### 1.1 Hyperion Imaging Mass Cytometry—a revolution in high multiplex imaging

**Fig. 1.**
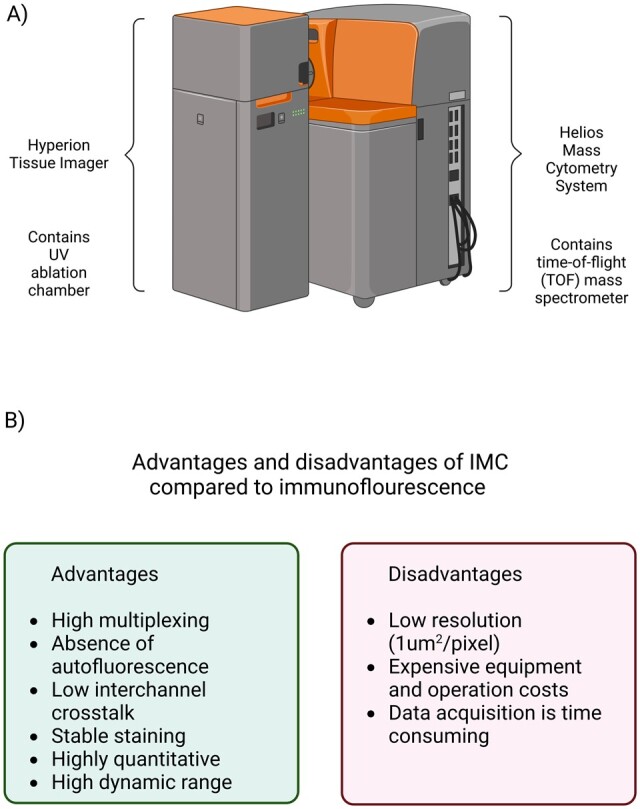
(**A**) A schematic representation of the Hyperion Imaging System. (**B**) The most important advantages and disadvantages of IMC compared to immunofluorescence (created with BioRender.com)

IMC was developed in 2014 based on earlier available suspension-based, single-cell mass cytometry [cytometry time of flight (CyTOF)] technology [described in Bandura *et al.* (2009)] but combined with an additional platform for UV ablation (Hyperion Tissue Imager, Standard BioTools, South San Francisco, USA) and performed on stained tissue sections, giving the spatial resolution of the data (Fig. 1A) (Bandura *et al.*, 2009). If we would draw a parallel between flow cytometry and mass cytometry techniques (CyTOF and IMC), the main difference would come to the fact that antibodies used for CyTOF and IMC are coupled with stable metal isotopes using metal-chelating polymer chains instead of fluorophores, and detection and quantification are performed in the time-of-flight (TOF) mass spectrometer (Helios mass cytometer), after which the data are exported into Flow Cytometry Standard (FCS) files for downstream analysis (Giesen *et al.*, 2014). Due to the high precision of the Helios mass cytometer in discriminating between isotope masses varying by only 1 Da, the IMC method at this point allows simultaneous detection of between 35 and 40 different markers with minimal crosstalk between the channels (Elaldi *et al.*, 2021; Guo *et al.*, 2020; Han *et al.*, 2017, 2018; Ijsselsteijn *et al.*, 2019) (Fig. 1B). This provides us with possibilities like never before to study in-depth histology and pathophysiology of the tissues, enables the discovery of new biomarkers, intercellular interactions and new cell microniches, and all that by using a rather simple workflow without a need for sequential staining of multiple sections. The method works well both with frozen and paraffin-embedded tissues (Guo *et al.*, 2020; Ijsselsteijn *et al.*, 2019).

**Fig. 2.**
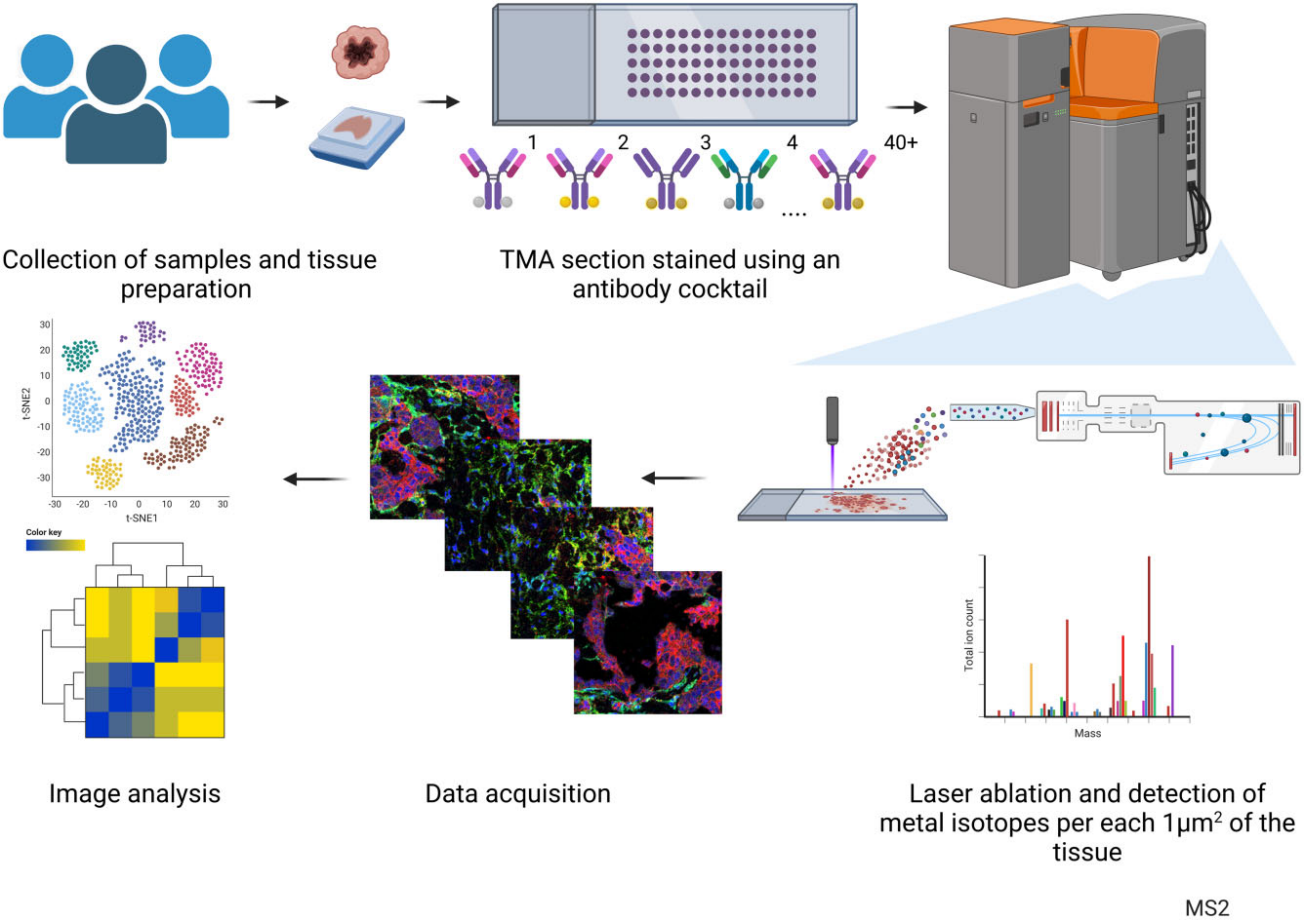
A schematic overview of the Imaging Mass Cytometry workflow (created with BioRender.com)

The data acquisition workflow is rather simple and begins with collecting and processing the tissue material to obtain either paraffin-embedded or frozen tissue sections. Tissue sections are first processed similarly as for IHC and then stained with a cocktail of metal isotope-coupled antibodies. Stained and dried tissue slides are loaded into the ablation chamber of the Hyperion tissue imager, where by using the mechanical system to move the tissue slide in a precise manner, stained tissues are ablated by a stationary UV laser beam (*λ* = 193 nm) 1 µm^2^ at a time. By each UV ablation cycle, 1 µm^2^ of the tissue is evaporated, creating plumes consisting of tissue and antibody residues, and metal isotopes. These plumes are being carried via argon gas into the CyTOF mass spectrometer (Helios mass cytometer, Standard BioTools, South San Francisco, USA), where the time of flight (TOF) is measured for each 1 µm^2^ of tissue, detecting the specific metal isotopes and their position on the grid, allowing the presence and position of each of the markers to be reconstructed creating a digital pseudo image of the tissue which is then used in image analysis (Fig. 2).

## 2 Raw data visualization and conversion

Visualization of complex imaging data is challenging, and as visual inspection of high multiplex imaging data is necessary for comprehending biological context, special software for IMC data visualization are needed (Windhager *et al.*, 2021). The raw data from IMC are in the MiniCAD Design File (MCD) format, consisting of listed signal intensities for each channel and signal position on the grid. This raw data format from the Hyperion instrument can be visualized using the MCD viewer (offered by Standard BioTools, South San Francisco, USA). This tool allows us to visualize the raw data in the image format, check for the quality of the staining in each of the channels, check for channel crosstalk and convert the raw data files into multichannel and single-channel .tiff files, suitable for downstream analysis. The most recent efforts in developing a package for multiplex data visualization in python utilize a multidimensional image viewer called napari (Windhager *et al.*, 2021) (https://zenodo.org/record/3555620). Napari utilizes a modular plugin called napari-imc to load raw IMC data into napari. Napari-imc is based on the readimc python package, previously developed by the Bodenmiller group for reading the raw IMC data (Windhager *et al.*, 2021).

Due to the high dimensionality of the IMC data, it is hard to comprehend the whole complexity of the images and draw scientific conclusions just by visually (qualitatively) evaluating the staining patterns. Therefore, in order to pool out the biological context behind the images and detected markers, images need to be subjected to complex analysis steps in order to obtain quantitative data on the spatial expression of markers of interest. Some of the available software and pipelines developed for and used on IMC data are summarized in the table below (Table 1).

**Table 1.** An overview of tools available for IMC data analysis

	IMC raw data visualization and/or conversion	Data pre-processing	Cell segmentation	Downstream analysis		
				Cell phenotyping	Spatial analysis	Pixel analysis
MCD Viewer	+	−	−	−	−	−

readimc (Windhager *et al.*, 2021)	+	−	−	−	−	−

Napari (using napari-imc plugin) (Windhager *et al.*, 2021)	+	−	−	−	−	−

histoCAT (Schapiro *et al.*, 2017)	−	−	−	+	+	−

MAUI (Baranski *et al.*, 2021)	−	+	−	−	−	−

IMC-Denoise (Lu *et al.*, 2023)	−	+	−	−	−	−

CellProfiler (Carpenter *et al.*, 2006)	−	+	+	+	+	–

DeepCell kiosk (Bannon *et al.*, 2021)	−	–	+	−	−	−

Cellpose (Stringer *et al.*, 2021)	−	−	+	−	−	−

IMC Segmentation Pipeline (Zanotelli and Bodenmiller, 2022)	+	+	+	−	−	−

Imcyto pipeline (Van Maldegem *et al.*, 2021)	−	−	+	−	−	−

MCMICRO (Schapiro *et al.*, 2022)	−	+	+	+	+	−

MATISSE (Baars *et al.*, 2021)	−	−	+	−	−	−

Dice-XMBD (Xiao *et al.*, 2021)	−	−	+	−	−	−

Steinbock (Windhager *et al.*, 2021)	+	+	+	−	−	−

RedSEA (Bai *et al.*, 2021)	−	+	−	−	−	−

Cytomapper (Eling *et al.*, 2020)	−	−	−	+	−	+

ImcRtools (Windhager *et al.*, 2021)	−	+	−	−	+	−

lisaClust (Patrick *et al*., 2023)	−	−	−	−	+	−

spicyR (Canete *et al.*, 2022)	−	−	−	−	+	−

Astir (Geuenich *et al*., 2021)	−	−	−	+	−	−

ACDC (Lee *et al*., 2017)	−	−	−	+	−	−

CELESTA (Zhang *et al*., 2022)	−	−	−	+	−	−

CellSighter (Amitay *et al.*, 2022)	−	−	−	+	−	−

Squidpy (Palla *et al.*, 2022)	+	−	+	−	+	−

SIMPLI (Bortolomeazzi *et al.*, 2022)	+	−	+	+	+	+

ImaCytE (Somarakis *et al.*, 2021)	−	−	−	+	+	−

CytoMAP (Stoltzfus *et al*., 2020)	−	−	−	+	+	−

SPEX (Pechuan-Jorge *et al.*, 2022)	−	+	+	+	+	−

Ilastik (Berg *et al*., 2019)	−	+	+	+	+	+

Almost all tools and pipelines discussed in this review (aside from MCD viewer and readimc) need .tiff files as an input, therefore an important step in IMC data analysis is a conversion of raw MCD data. For data conversion, readimc python package is most often being used, although MCD viewer has the capability of exporting single-channel and multi-channel .ome.tiff files. As already stated, the readimc python package is being implemented in the napari-imc plugin but is also part of the IMC Segmentation Pipeline and Steinbock framework, as its integral file conversion step (discussed further down). The only tools discussed in this review that can be used exclusively for IMC data are readimc and MCD viewer. All other tools can read .tiff files and have the potential to be implemented in the analysis of data acquired by other high multiplexed imaging technologies (e.g. Codex) (Table 2).

**Table 2.** Utility of available tools

	Interoperability with other imaging platforms	Platform	Year of release	Estimation of bioinformatic skills level required	Advantages	Disadvantages
MCD Viewer	−	GUI	2017	Basic	User-friendly interface Easy to use	Available only for Windows OS

readimc (Windhager *et al.*, 2021)	−	Python	2021	Medium	Implemented in a number of other IMC tools	Command line only

Napari (using napari-imc plugin) (Windhager *et al.*, 2021)	−	GUI/Python	2021	Medium	Data visualization tool suitable for all OS Easy to use	Accessible from the command line only

histoCAT (Schapiro *et al.*, 2017)	+	GUI/MATLAB	2017	Basic	User-friendly interface Easy to use	Poor scalability

MAUI (Baranski *et al.*, 2021)	+	GUI/MATLAB	2021	Expert	Capable of removing speckles	Subjective and labor-intensive

IMC-Denoise (Lu *et al.*, 2023)	+	Jupyter Notebook	2022	Expert	Fully automated Requires minimal input	Requires strong hardware configuration Cannot remove speckles

CellProfiler (Carpenter *et al.*, 2006)	+	GUI/Python	2005	Medium	User-friendly interface Flexible	Requires operator’s input

DeepCell kiosk (Bannon *et al.*, 2021)	+	Google Cloud/Shell	2021	Basic	High scalability Good performance	Requires the use of the command line

Cellpose (Stringer *et al.*, 2021)	+	GUI/Jupiter Notebook	2020	Basic	High scalability Trained and constantly retrained on large and variable datasets	Requires the use of the command line to be set up and run locally

IMC Segmentation Pipeline (Zanotelli and Bodenmiller, 2022)	+	GUI/Python	2020	Medium	With direct input from the experienced operator, it offers more control over the segmentation process	Involves multiple steps Time consuming compared to other methods Sensitive to low resolution of IMC data

Imcyto pipeline (van Maldegem *et al.*, 2021)	+	Nextflow/Python	2020	Medium	Compact, portable and reproducible	Involves multiple steps Requires operator’s input

MCMICRO (Schapiro *et al.*, 2022)	+	GUI/Nextflow/Python	2022	Basic	User-friendly interface Ability to perform multiple image analysis tasks	Requires operator’s input
MATISSE (Baars *et al.*, 2021)	+	GUI/R/Python	2020	Medium	DAPI staining improves segmentation by bypassing issues of IMC data low resolution	Involves multiple steps Requires operator input Time consuming compared to other methods

Dice-XMBD (Xiao *et al.*, 2021)	+	Python	2021	Expert	Designed specifically for IMC data High accuracy in cell segmentation	Requires bioinformatic background and experience in using python

Steinbock (Windhager *et al.*, 2021)	+	Python/Dockerfile	2021	Basic	Simple to use High accuracy in cell segmentation Prepares data for further analysis steps	Command line only

RedSEA (Bai *et al.*, 2021)	+	MATLAB/Jupyter Notebook	2021	Expert	Successfully removes lateral spillover and assigns the signal to the correct source	Cannot correct for spillover caused by overlapping cells Its effect needs to be controlled by experienced operator

Cytomapper (Eling *et al.*, 2020)	+	R	2020	Medium	Suitable tool for cell segmentation quality control High scalability	Requires R skills Command line only

ImcRtools (Windhager *et al.*, 2021)	+	R	2020	Medium	High range of possibilities for IMC data analysis High scalability	Requires R skills Command line only

lisaClust (Patrick *et al.*, 2023)	+	R	2021	Medium	Performs spatial analysis using local indicators of spatial associations	Requires R skills Command line only

spicyR (Canete *et al.*, 2022)	+	R	2022	Medium	Useful in identification of subtle differences in spatial composition across different samples	Requires R skills Command line only

Astir (Geuenich *et al.*, 2021)	+	Python	2021	Expert	Scalable Supervised and adaptable cell classification tool	Requires an input from experienced operator Command line only

ACDC (Lee *et al.*, 2017)	+	Python	2017	Expert	Semisupervised approach for cell classification Allows discovery of novel cell populations	Requires experience in using python Command line only Not originally designed for digital image data
CELESTA (Zhang *et al.*, 2022)	+	R	2022	Expert	Takes into consideration tissue structure when assigning cell classes	Requires input from an experienced operator Command line only

CellSighter (Amitay *et al.*, 2022)	+	Python	2022	Expert	High accuracy and precision in assigning cell classes Reduced data overfitting	Requires input from an experienced operator Requires experience in using python

Squidpy (Palla *et al.*, 2022)	+	Python	2022	Expert	High level of freedom working with data High scalability	Requires experience in using python Not originally designed for digital image data

SIMPLI (Bortolomeazzi *et al.*, 2022)	+	Nexflow	2021	Basic	Ability to perform multiple image analysis tasks	Not flexible

ImaCytE (Somarakis *et al.*, 2021)	+	GUI/Matlab	2019	Basic	User-friendly interface Implemented niche discovery	Poor Scalability

SPEX (Pechuan-Jorge *et al.*, 2022)	+	GUI/modular	2022	Basic	Modular Interactive All-in-one tool	Not flexible

CytoMAP (Stoltzfus *et al.*, 2020)	+	GUI/MATLAB	2020	Medium	User-friendly interface	Poor Scalability

Ilastik (Berg *et al.*, 2019)	+	GUI/Python	2019	Basic	Interactive Intuitive	Requires at least basic knowledge of histology for correct annotations

## 3 Data preprocessing

Although IMC is unaffected by autofluorescence and signal spillover, common in IF-based imaging techniques (Fig. 1B), in some instances, some of the markers may exhibit weak signal, low signal-to-noise ratio, channel crosstalk, background noise and artifacts in pixel intensity (e.g. hot pixels and speckles) (Baranski *et al.*, 2021; Chevrier *et al.*, 2018; Ijsselsteijn *et al.*, 2021; Lu *et al.*, 2023). Hot pixels are the most common artifacts detected in the IMC images, likely caused by detector abnormalities. They are represented as individual pixels with unusually higher signal intensities (high counts of ions) when compared to surrounding pixels. Speckles are larger areas of high signal intensity that do not correspond to biological structures, and which most probably occur due to the unspecific binding and antibody aggregates, or possibly due to contamination with dust particles (Baranski *et al.*, 2021; Xiao *et al.*, 2021).

The signal spillover can be addressed using the pre-acquisition methods, e.g. CATALYST compensation workflow (Chevrier *et al.*, 2018). Here the signal compensation matrix is created, and signal compensation is performed using the CATALYST R/Bioconductor package (explained further below). Other ways to assess the problem of channel crosstalk are post-acquisition methods such as signal compensation [described in work by Wang *et al.* (2019)]. Nevertheless, channel crosstalk is usually of low intensity and doesn’t always interfere with the downstream analysis (Lu *et al.*, 2023).

Background noise is defined as an unwanted variation in the image information. It can be addressed using ilastik (Berg *et al.*, 2019) for background noise reduction, as described in work by Ijsselsteijn *et al.* (2021). Here ilastik pixel classification is used to define which pixels belong to the actual signal and which pixels belong to the background noise. This is done for each marker where an experienced operator would train the forest pixel classifier by manually annotating pixels that either belong to the actual signal or the background. ilastik random forest pixel classifier, trained based on information received from the operator’s input, classifies each pixel either as a signal or background. The output is then in the form of binary expression maps, where the ‘background’ pixels will be set to the value 0 and the ‘signal’ pixels to the value 1 (Ijsselsteijn *et al.*, 2021). Sometimes the differences in staining intensities can be due to the sample batch effect, which is an unwanted variation in signal intensities of a given channel between different samples. The batch effect arises from having tissue samples processed in a different manner, which can cause different reactivity of tissues to used antibodies, giving uneven signal intensity across the samples (Somarakis *et al.*, 2021). Batch effect and uneven signal intensities across different samples can affect downstream analysis, and these issues need to be addressed before proceeding with the subsequent analysis steps. Images binarized in this way, level up the signal across all the samples, consequently removing the batch effect issues. In further analysis, the single-cell profiles are then assessed by counting the positive signal frequency and not assessing the mean signal intensity per cell (Ijsselsteijn *et al.*, 2021; Lu *et al.*, 2023). The ilastik signal binarization method indeed works exceptionally well in removing background noise but also in unifying and normalizing signals across samples eliminating the batch effect.

Due to the importance of having a corrected, artifact-free signal for the downstream analysis of the IMC data, several pipelines have recently been developed to deal solely with IMC data preprocessing.

### 3.1 CATALYST channel crosstalk correction

IMC, in general, does not have issues with inter-channel crosstalk. Nevertheless, a small degree of crosstalk can occur in the adjacent mass channels due to the presence of trace variant isotope contaminants in the metal solutions used in conjugation, chemical reactions of the metals or due to imprecision in isotope detection. CATALYST pipeline has been designed by the Bodenmiller group in order to generate the spillover matrix, which is then used to correct for crosstalk in adjacent channels (Chevrier *et al.*, 2018). The spillover matrix is created on the base of signals detectable in adjacent channels, acquired by the Hyperion system on each separate antibody conjugate (Chevrier *et al.*, 2018). Although this method works well, preparations of the agarose glass slide for this purpose can be time consuming and labour intensive. A detailed explanation of the protocol is available at https://bodenmillergroup.github.io/IMCDataAnalysis/spillover-correction.html.

### 3.2 MAUI for IMC data preprocessing

Another approach for high multiplex data preprocessing has been described in a paper by Baranski *et al.* Here, the authors presented MAUI (MBI Analysis User Interface), MATLAB-based software primarily developed for MIBI data but has also been shown to work well on IMC data for the removal of aggregates (speckles), signal crosstalk and background noise reduction (Baranski *et al.*, 2021).

Aggregates removal in MAUI is based on the detection of small continuous pixels with positive ion counts that, due to their small size, cannot represent biological structures. The first step is to blur images using a Gaussian filter, this causes larger patches of pixels in close proximity to merge. Using the software interface, the operator can set the size threshold in order to eliminate (transform pixel values to 0) the objects considered to be artifacts.

To tackle the problem of signal crosstalk from adjacent channels, the MAUI uses a specifically designed pipeline divided into two main steps. In the first step, the contaminating channel is processed by capping, smoothing and binarizing the signal in order to get the binary masks (signal positive pixels get value 1 and signal negative pixels get value 0) (Baranski *et al.*, 2021). In the second step, the binary masks are used to clean images in other channels, which is performed by subtracting a fixed value from all the pixels in the target channel that were positive in the contaminant mask (Baranski *et al.*, 2021).

Background removal in MAUI works on deionizing algorithm following the k-Nearest Neighbor (k-NN) approach (Mastin, 1985). This pipeline is divided into two phases. In the first phase, the density count near each pixel is determined by calculating the distance from the certain pixel to the closest k non-zero pixels (Baranski *et al.*, 2021; Mastin, 1985). In the second phase, the signal coming from pixels with low-density ion counts is filtered out by choosing the threshold for separating noise and signal (Baranski *et al.*, 2021). This approach works based on the assumption that noise signals are more equally scattered than the real signal (Baranski *et al.*, 2021).

MAUI is a useful pipeline for IMC data preprocessing, which integrates hot pixel, background noise and crosstalk correction. Although the authors report successful results in using this pipeline, it requires constant input from an experienced operator, can be subjective and is labor intensive. In addition to being labor intensive, the main limitation of this method lies in the fact that set threshold values dividing signal from the noise are overly subjective and could largely vary between the channels and images (tissue samples). A more detailed description of this pipeline, together with the code and the user manual, is available at https://github.com/angelolab/MAUI.

### 3.3 IMC-Denoise

The most recent pipeline published on IMC data preprocessing is a methodology paper by Lu *et al.* (2023), where the authors presented IMC-Denoise, a content-aware pipeline for enhancing IMC data. In their work, they used a differential intensity map-based restoration algorithm (DIMR) in order to first remove ‘hot’ pixels, and a self-supervised deep learning algorithm for filtering out the noise (DeepSNF) (Lu *et al.*, 2023).

The authors reported IMC-Denoise performing better in preprocessing IMC images for background removal and signal binarization than single threshold binarization, semi-automated ilastik background removal and MAUI, evaluating the results by using the F1 score as an accuracy metric (Lu *et al.*, 2023). The superiority of this pipeline was also residing in the fact that it was fully automated, demanding minimal operator input. DeepSNF was able to distinguish well between the noise and the signal and enabled improved sensitivity and specificity in cell clustering and cell phenotyping in the downstream analysis when compared with data preprocessed using previously described methods (Lu *et al.*, 2023). The DIMR algorithm was shown to be very successful in removing single hot pixels and small size speckles (Lu *et al.*, 2023). This pipeline is therefore limited when applied to removing hot pixels forming larger clusters because the DIMR algorithm cannot discriminate artifacts from the real signal in larger areas (Lu *et al.*, 2023). A detailed description of the algorithms used in this pipeline is available in the original study by Lu *et al.* (2023), and the code source, together with the user tutorial, is available at https://github.com/PENGLU-WashU/IMC_Denoise.

## 4 Cell segmentation

Cell segmentation is the first and maybe the most critical step in digital image analysis. To make a biological interpretation of the IMC images, the meaning behind each of the pixels we see on our screen needs to be given. This is accomplished by performing cell segmentation, where by using different approaches, we define certain areas and certain pixels in the image as defined objects, cells or subcellular compartments (e.g. nucleus, cytoplasm and membrane). It is important to approach this step with care and choose the approach that works the best on subjected images, as the whole downstream steps and the accuracy of the subsequently obtained data depend directly on the quality and accuracy of cell segmentation. Cell segmentation methods can be roughly divided into two groups (Chen and Murphy, 2022). The first group represents geometry-based techniques such as region and threshold-based segmentation (Panagiotakis and Argyros, 2018; Shen *et al.*, 2018), watershed segmentation (Ji *et al.*, 2015), Chan-Vese segmentation (Braiki *et al.*, 2020), active contour (Wu *et al.*, 2015) and Graph-cut based segmentation (Oyebode and Tapamo, 2016). In the second group are more advanced techniques based on deep convolutional neural networks (CNN) (Jung *et al.*, 2019; Sadanandan *et al.*, 2017). Particularly are in use U-Net (Al-Kofahi *et al.*, 2018; Falk *et al.*, 2019; Long, 2020; Ronneberger *et al.*, 2015), DeepCell (Van Valen *et al.*, 2016) and Mask R-CNN (Fujita and Han, 2021; Johnson, 2018; Lv *et al.*, 2019), as well as ensemble methods designed to utilize multiple deep learning platforms (Baykal Kablan *et al.*, 2020; Vuola *et al.*, 2019).

### 4.1 Bodenmiller cell segmentation pipeline (IMCSegmentationPipeline)

Bodenmiller cell segmentation pipeline has been developed in the Bernd Bodenmiller group to be used specifically on IMC data [steps presented in detail in https://bodenmillergroup.github.io/ImcSegmentationPipeline, (Zanotelli and Bodenmiller, 2022)]. In short, this pipeline utilizes ilastik (Berg *et al.*, 2019) for pixel classification and CellProfiler (Kamentsky *et al.*, 2011; Baars *et al.*, 2021; Preim and Botha, 2014; Stirling *et al.*, 2021; Vincent and Soille, 1991) for watershed segmentation (Carpenter *et al.*, 2006; Zanotelli and Bodenmiller, 2022) of ilastik derived probability maps.

This method works exceptionally well, with a drawback that it requires multiple steps to be performed using different software packages. It can be more time consuming than other approaches and requires the expertise of operators both in histology and bioinformatics. It is originally based on nuclear staining, meaning the information on complex cell morphology could be missed. The relatively low resolution of the IMC data (1 µm^2^/pixel) can be challenging for this approach to distinguish between cells in more densely packed regions (e.g. discrimination between cancer cells and infiltrated lymphocytes in the epithelial compartment) (Baars *et al.*, 2021). In addition, watershed segmentation can be unpredictable as it is based on the intensity of individual pixels, when a single hot pixel can significantly alter the segmentation outcome (Jones *et al.*, 2005). These deficiencies are being attempted to resolve by using combination of IMC and DAPI nuclear staining (see Section 4.5).

### 4.2 Cell segmentation using Steinbock

Steinbock is a framework for a dockerized version of the previously described IMCSegmentationPipeline (Windhager *et al.*, 2021). Steinbock simplifies IMC raw data processing, encompassing steps such as data pre-processing, cell segmentation and feature extraction into an easy-to-use command line interface (Windhager *et al.*, 2021). To perform cell segmentation, aside from the random forest classifier-based approach described earlier, Steinbok also supports a deep learning cell segmentation approach based on DeepCell architecture (Greenwald *et al*., 2022). In short, user-defined image channels are first combined to create two channel-image consisting of a nuclear and cytoplasmic signal. Then, the DeepCell python package is used to run a pretrained network (pretrained on TissueNet data) called Messmer to generate cell masks (Windhager *et al.*, 2021).

After cell segmentation, Steinbock quantifies features of detected objects such as area, eccentricity, spatial neighbors and marker intensities (Windhager *et al.*, 2021). These measurements contain useful information needed for downstream analysis. Data extracted from Steinbock can then be directly used with the imcRtools R/Bioconductor package for both data visualization and spatial analysis(described in more detail below) (Amezquita *et al.*, 2020; Righelli *et al.*, 2022; Schapiro *et al.*, 2017; Windhager *et al.*, 2021).

### 4.3 Nf-core/imcyto pipeline

Nf-core/imcyto is an automated, Nextflow-based implementation of Bodenmiller’s cell segmentation pipeline [https://github.com/nf-core/imcyto (van Maldegem *et al.*, 2021)]. It is written using the Nextflow workflow tool, which has the ability to be run on multiple computer platforms and supports container technologies such as docker and singularity (van Maldegem *et al.*, 2021). This ensures high interoperability and reproducibility across different computing platforms and institutions, and helps in combining and implementing different workflows based on different scripting languages (Ewels *et al.*, 2020). Imcyto pipeline combines singularity or docker containers for readimc, CellProfiler and ilastik into a single portable and reproducible pipeline for cell segmentation (van Maldegem *et al.*, 2021). The input files are raw .mcd or .txt files, or ome.tiff image files, together with channel and marker binary information in the form of a .csv file. Additional files needed for the pipeline to operate are pre-trained ilastik project files (.ilp) and CellProfiler pipeline files (.cppipe). Aside from output files in the form of probability maps, cell masks and .csv files containing cell relationships and single measurements, this pipeline will also generate a report of pipeline performance (van Maldegem *et al.*, 2021).

### 4.4 MCMICRO (Multiple-choice microscopy pipeline)

MCMICRO is modular, another Nextflow-implemented pipeline developed for automated image analysis of large microscopy data [https://github.com/labsyspharm/mcmicro (Schapiro *et al.*, 2022)]. It has the capability of performing multiple tasks, such as illumination correction of light in IF microscopy images, image stitching and registration, cell segmentation, and finally, cell phenotyping.

The most relevant use of MCMICRO for IMC data is cell segmentation. To perform cell segmentation, MCMICRO employs the deep learning algorithm UnMICST (U-Net model for identifying cells and segmenting tissue) (Yapp *et al*., 2022). This is a preprocessing module in MCMICRO that helps in increasing the accuracy of cell segmentation by creating pixel class probability maps (Schapiro *et al.*, 2022). This pipeline also relies on ilastik for the same purpose of generating pixel probability maps using the random forest classifier. Probability maps obtained by UnMICST and ilastik are subjected to watershed segmentation via S3segmenter. S3segmenter is a MATLAB-based watershed algorithm that generates single-cell masks from the probability maps obtained in the previous step (Schapiro *et al.*, 2022).

### 4.5 MATISSE (iMaging mass cytometry mIcroscopy Single-cell SegmEntation)

MATISSE has been developed particularly to address challenges in IMC data segmentation that arise due to low resolution. It has been designed to utilize IMC-derived signal for cytoplasmic, nuclear and membrane staining, but also fluorescent DAPI staining of the same tissue section [https://github.com/VercoulenLab/MATISSE-Pipeline (Baars *et al.*, 2021)]. Two types of image data acquired using IF and IMC can be overlayed based on the nuclear DAPI/Ir staining pattern. Pixel classification is then performed on DAPI staining by an experienced operator using ilastik to generate probability maps, which are then used in CellProfiler to generate cell masks using watershed algorithm. Due to the higher resolution of data used for pixel classification (DAPI nuclear staining), this approach overperformed the classical Bodenmiller ilastik/CellProfiler segmentation pipeline described above (Baars *et al.*, 2021).

### 4.6 Deep learning-based segmentation tools

The rapid advancement of deep learning has led to the development of many tools that could be successfully applied to cell segmentation (Araújo *et al.*, 2019; Tran *et al.*, 2018). Some of the deep learning-based tools available for cell segmentation are DeepCell, Cellpose, StarDist, Nuclealzer, Piximi, ImJoy, etc. (Hollandi *et al.*, 2020; Lucas *et al.*, 2021; Ouyang *et al.*, 2019; Schmidt *et al.*, 2018; Stringer *et al.*, 2021; Van Valen *et al.*, 2016). Segmentation techniques based on deep learning can be divided into semantic and instance segmentation. Semantic segmentation is segmenting the images into semantically similar pixel groups, assigning each pixel class as nuclear, cytoplasmic or background producing a pixel-level classification and not necessarily containing the object information (Moen *et al*., 2019). Examples of these are the U-Net algorithm (Ronneberger *et al.*, 2015) and the convolutional neural networks (CNN) algorithm (Din and Yu, 2021). Instance segmentation, on the other side, identifies each distinct object class, containing distinct object information (Moen *et al.*, 2019). An example of this is Mask-RCNN (He *et al.*, 2017). Among the palette of models based on deep learning that has been developed in the past years for cell segmentation, the most in use for IMC data and the most accurate are U-NET and CNN algorithms (Chen and Murphy, 2022).

#### 4.6.1 Dice-XMBD

Dice-XMBD is a cell segmentation pipeline designed specifically for IMC data (Xiao *et al.*, 2021). The dice-XMBD pixel classification model is based on combining ilastik operator-driven pixel classification and U-Net deep learning model to obtain a generic pixel classifier, which can be utilized for classification of individual pixels to their distinct cellular compartment of origin (membrane/cytoplasm, nuclei and intercellular space). This classification process results in generating probability maps that are used as an input file for CellProfiler, which uses watershed segmentation pipeline for generation of cell masks (Xiao *et al.*, 2021).

The main strength of this approach is that it is able to get around the general limitations reflected in training data scarcity and achieve a human-level cell segmentation performance by combining the effectiveness of the already well-proven U-Net algorithm, and applying the refined expert knowledge input through ilastik as a teacher model [(Xiao *et al.*, 2021) https://github.com/xmuyulab/Dice-XMBD].

#### 4.6.2 Cellpose

Cellpose is a generalist cell segmentation algorithm based on U-Net and trained on a large dataset consisting of various types of micrographs, as well as non-microscopic images consisting of a big number of repeating objects (fruits, snail shells, etc.) [https://github.com/MouseLand/cellpose (Stringer *et al.*, 2021)]. The cellpose algorithm is constantly being re-trained by a big number of everyday users, further improving the algorithm and contributing to its performance. The designers of Cellpose evaluated its performances compared to manually annotated images, used as the ground truth, as well as to other state-of-the-art deep learning segmentation algorithms, such as Mask R-CNN [https://github.com/dpeerlab/MaskRCNN_cell (He *et al.*, 2017)], StarDist [https://github.com/stardist/stardist (Schmidt *et al.*, 2018)] and class two and three U-Net networks (https://github.com/PARMAGroup/UNet-Instance-Cell-Segmentation). The authors concluded that Cellpose was able to outperform all other compared algorithms (Stringer *et al.*, 2021).

#### 4.6.3 DeepCell

DeepCell is a CNN-based tool designed for the accurate segmentation of bacterial and mammalian cells (Van Valen *et al.*, 2016). The deep CNN algorithm is trained to perform three distinguished segmentation tasks. It has been trained on phase microscopy images of bacteria, in order to perform segmentation of bacterial cytoplasm, on IF microscopy images of the mammalian nuclear staining, in order to be able to perform nuclear segmentation of mammalian cells, and on phase microscopy images of mammalian cells and IF images of nuclear staining in order to perform segmentation of mammalian cell cytoplasm (Van Valen *et al.*, 2016).

DeepCell Kiosk is a DeepCell-based software package developed as an open-access source aiding in cell segmentation to an entire community of researchers working with image analysis (Bannon *et al.*, 2021). It utilizes clusters on Google Kubernetes Engine (GKE) to run the DeepCell and is accessible through the web portal. GKE clusters are fed data through either an ImageJ plugin or using front end command line tool (Bannon *et al.*, 2021). Uploaded data are kept in a database accessible to the segmentation pipeline, where the cell segmentation can be performed, and then the output files can be downloaded from the cloud. DeepCell Kiosk allows users to process a big amount of data in a time-efficient and cost-effective way, bringing the utility of deep learning in image analysis closer and more accessible (Bannon *et al.*, 2021).

### 4.7 Cell segmentation quality control

Whichever of the proposed methods for cell segmentation is being used, it is advisable to check if the cell segmentation is done correctly by overlaying cell masks with specific staining (nuclear, cytoplasmic or membrane staining). That is a very important step in obtaining the data's correctness and assuring that downstream analysis is being performed accurately, avoiding biases. This task is supported by the cytomapper R/Bioconductor package, which enables a visual assessment of obtained cell masks by outlining cell objects over composite images showing the channels (markers) of interest and staining patterns (Eling *et al.*, 2020).

Even when segmentation is done flawlessly, lateral signal spillover may occur in the cases of adjacent cells (cells in very close proximity). This can cause errors in cell phenotyping, manifesting in biologically implausible marker combinations. This imperfection can be addressed using either whole cell or border signal compensation, or applying the RedDSEA algorithm for automated lateral spillover compensation. The later has been proven superior in comparison with signal subtraction (compensation) methods (Bai *et al.*, 2021); therefore, it will be presented here in more detail.

### 4.8 Lateral spillover compensation—RedDSEA (REinforcement Dynamic Spillover EliminAtion)

Even when the cell segmentation is performed flawlessly, problems in the downstream analysis, particularly in the single-cell phenotyping, could arise from lateral signal spillover. This means that the signal from one cell object can be detected in the adjacent cell object due to the strong and excessive signal in the channel, the close proximity of cells in densely packed regions, and due to interleaving and physical overlapping of cell membranes and cytoplasm of adjacent individual cells (Goltsev *et al.*, 2018; Jackson *et al.*, 2020). This results in getting ambiguous, biologically implausible marker expression patterns such as, e.g. CD3/CD20 positive cell populations (see Jackson *et al.*, 2020), or CD3/Pancytokeratin (PanCk) positive cell populations. To address this problem, Bai *et al.* (2021) designed a spillover compensation algorithm named RedDSEA. As in the biblical story of Moses dividing the Red Sea, this approach helps in dividing the signal between adjacent cells. The RedDSEA algorithm calculates the percentage of the shared border between adjacent cells, and then it corrects the signal focusing only on the pixels near the adjacent cell borders. This algorithm also has the capability to correctly assign the spillover signal to the correct cell of origin, additionally helping in the classification of cells with low signal abundance, by reinforcing the weak signal and assigning it to the correct cell object (Bai *et al.*, 2021). RedDSEA works independently of the operator’s intervention and requires only multichannel .tiff input and the cell mask. It is a great tool for minimizing the number of mismatched cell classes by improving the precision of single-cell phenotyping, and reducing the misinterpretation of downstream analysis data (Bai *et al.*, 2021). Some limitations of RedSEA are that it cannot correct for the spillover in the case of overlapping cells, in the case of channel crosstalk and image artifacts. Additionally, it cannot compensate for improper cell segmentation and therefore its performance directly depends on the quality of cell segmentation (Bai *et al.*, 2021). Although RedSEA works well in correcting the lateral spillover in an unsupervised manner, it should be used cautiously, its effects should be controlled based on the predefined knowledge and the signal should be corrected only in the channels where it is necessary (Bai *et al.*, 2021).

## 5 Downstream analysis

Which software and methods will be used in the downstream analysis largely depends on the research questions one wants to answer. The common steps in analyzing IMC data after cell segmentation are cell clustering, cell phenotyping and dimensionality reduction followed by differential analysis, and spatial analysis (Windhager *et al.*, 2021).

### 5.1 Bioconductor

Bioconductor is an open-source, R-based project designed for the distribution of packages developed by a large and diverse community of users for the analysis of high-throughput genomic and molecular biology data (Amezquita *et al.*, 2020; Gentleman *et al.*, 2004; Huber *et al.*, 2015; Lawrence *et al.*, 2013; Robinson *et al.*, 2010; Schürch *et al.*, 2020). From the perspective of IMC data, the Bodenmiller group has developed two Bioconductor packages that help perform the downstream analysis. These tools are imcRtools (https://github.com/BodenmillerGroup/imcRtools) and Cytomapper (https://github.com/BodenmillerGroup/cytomapper) (Windhager *et al.*, 2021).

Cytomapper uses multichannel .tiff images and cell masks as input data to visualize the spatial data and to perform single-cell spatial data analysis. It helps in performing cell segmentation quality control by visualizing the cell outlines directly in R and overlying cell masks on images with specific staining. It provides a possibility for visualizing specific cell types and their spatial distribution in an image as well as outlining defined specific cell types and overlaying cell outlines with markers of interest, which can be used as a quality control of cell phenotyping. It provides a possibility of gating, giving an interactive environment where one can follow the quality of cell phenotyping in real time using this option by controlling the defined cell population identity directly in the image, judging the marker expression, segmentation quality and potential lateral spillover that could all influence errors in the cell classification process. This establishes the ground truth base for further cell classification (Eling *et al.*, 2020).

**Fig. 3.**
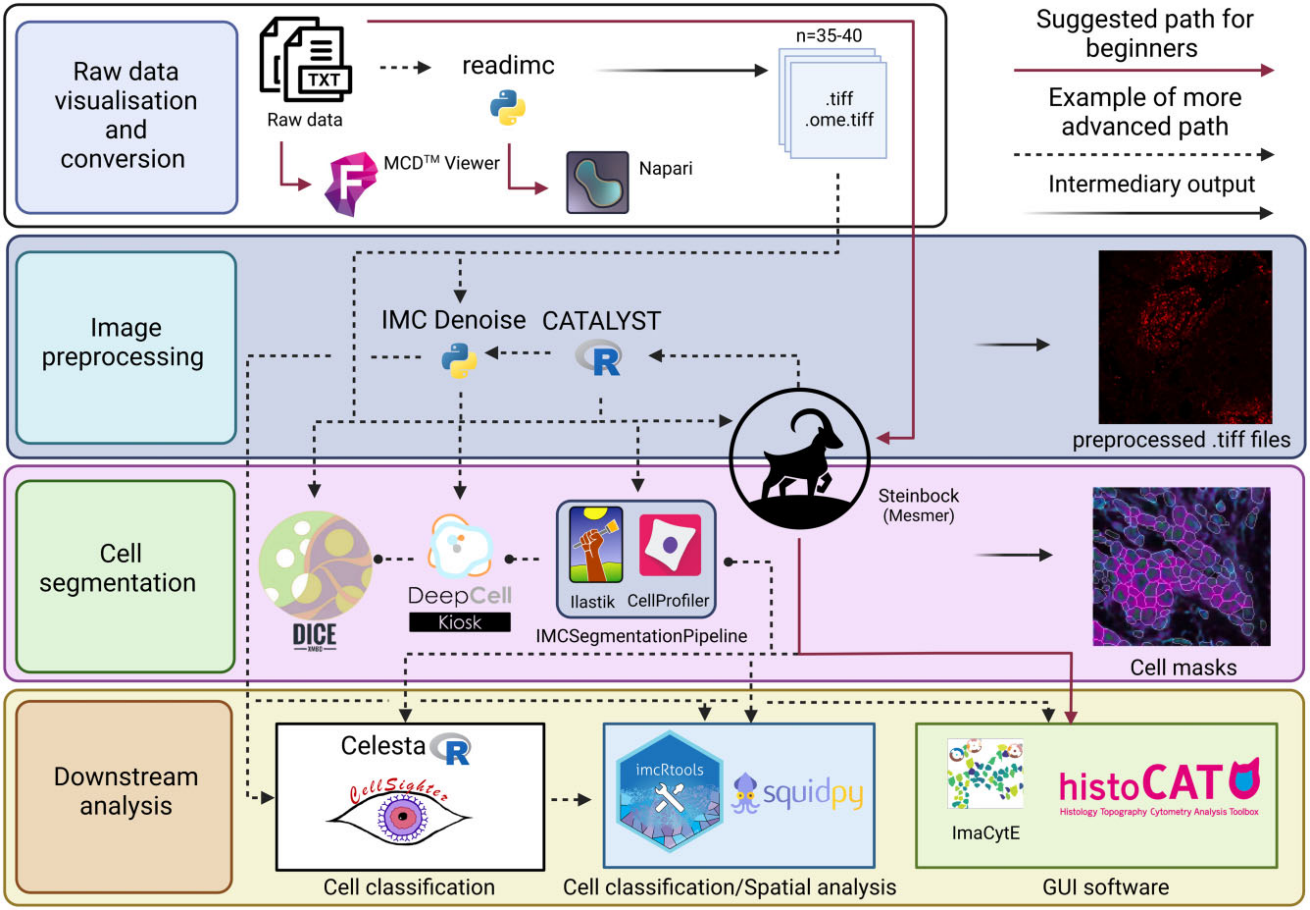
Examples of possible IMC analysis workflows. Raw data in the form of.txt files are visualized using either MCD Viewer or napari-imc plugin, to evaluate the staining quality. Steinbock framework performs image pre-processing, cell segmentation and single-cell data extraction using simple commands, and therefore is a suitable tool for beginners. Images acquired using e.g. Steinbock, can be pre-processed using the CATALYST pipeline and used in further analysis, or further pre-processed using the IMC Denoise pipeline. Pre-processed images could be utilized for cell segmentation or directly used in downstream analysis. Outputs obtained by Steinbock are possible to use instantly by imcRtools or Squidpy packages. An alternative approach would be to first perform cell classification using Celesta or CellSighter before undertaking spatial analysis in imcRtools or Squidpy. For beginners, data obtained from Steinbock can be read by ImaCytE and histoCAT for simple analysis (created with BioRender.com)

ImcRtools is another package designed by the Bodenmiller group for spatial analysis of IMC data. Input files used are the standard output files from Steinbock and IMC Segmentation Pipeline, providing simplicity in data handling and bridging image preprocessing and segmentation tools and downstream image analysis strategies offered by Bioconductor (Fig. 3) (Windhager *et al.*, 2021). ImcRtools offers robust possibilities for spatial analysis. Using imcRtools, we can firstly visualize spatial interaction graphs (used as a base for spatial analysis), perform spatial community detection [regions defined based on the spatial proximity of cells, as e.g. epithelial/stromal communities, proposed by Jackson *et al.* (2020)], perform cellular neighborhood analysis [localized regions with specific cell type composition, first proposed by Goltsev *et al.* (2018) and Schürch *et al.* (2020)], spatial context analysis [spatial context refers to specific regions in the tissue where defined cell neighborhoods may interact (Bhate *et al.*, 2022)], perform patch detection [a supervised approach for detection of interconnected groups of similar cell classes cells, proposed by Hoch *et al.* (2022) and cell–cell interaction analysis proposed by Schapiro *et al.* (2017) and implemented in histoCAT].

In addition, lisaClust and SpicyR packages have been recently developed and are available at Bioconductor for spatial analysis (Patrick *et al.*, 2023; Canete *et al.*, 2022). Compared to imcRtools, lisaClust offers an alternative approach to cellular neighborhood analysis (Patrick *et al.*, 2023). LisaClust utilizes local indicators of spatial associations (LISA) function on the SegmentedCells object, previously generated by SpicyR (Patrick *et al.*, 2023; Canete *et al.*, 2022), to compute LISA curves across different radial distances from the cells, based on which it performs clustering to characterize cell interactions between different cell classes (Patrick *et al.*, 2023).

SpicyR is a useful tool for differential analysis of spatial cell localization across different images and is a powerful tool for identifying subtle differences in cellular composition across different samples. It has demonstrated an ability to summarize differences in cell localizations across different images and associated these microenvironment variations with different pathological states (Canete *et al.*, 2022).

### 5.2 Astir (ASsignmenT of sIngle-cell pRoteomics) cell annotator

Astir is a scalable, probabilistic method that uses deep recognition neural networks and input from the operator in the form of predefined knowledge of marker expression patterns in specific, *a priori* known and expected cell classes to perform cell phenotyping in the supervised, hierarchical manner [https://www.github.com/camlab-bioml/astir, (Geuenich *et al.*, 2021)]. The main statistical model used in Astir relies on the assumption that the cell phenotype is a static and unchanged pattern of expression of certain markers and lack of expression of others. In addition, the probability of a certain cell belonging to a certain phenotype is also based on the assumption that such specific cells will have significantly higher expression of key markers than the other cells belonging to other phenotypes. It is also taken into consideration that high expression of key markers can vary to a certain degree for the same cell phenotype. It also allows the detection of cells that, by their marker expression, do not fit into any of *a priori* set categories, allowing the identification of new ‘unknown’ cell phenotypes (Geuenich *et al.*, 2021).

Astir is the first tool of this type developed for systemic and adaptable cell phenotyping and the only tool so far that offers flexibility in the level of certainty when defining cell classes, giving a user possibility to specify the probability threshold for marker expression at which some cells will be considered of ‘unknown’ or ‘other’ phenotype. Cells that are assigned as ‘unknown’ are cell populations that do not belong to any of the groups of cells expressing *a priori* known pattern of cell markers and could possibly represent a novel population of cells (Geuenich *et al.*, 2021).

The downside of this method is that Astir takes into consideration only marker expression patterns but not the tissue architecture and spatial orientation of cells, which is as well an important feature. Nevertheless, according to its designers, Astir performed better when compared to similar available software (e.g. FlowSOM, PhenoGraph and ClusterX) in accuracy and robustness (Geuenich *et al.*, 2021).

Software similar to Astir is Garnett, initially developed by the Trapnell group, for automated single-cell annotation based on single-cell sequencing data (Pliner *et al*., 2019). Although it has the potential to be also used for IMC data analysis, so far it has not been in extensive use for this purpose.

### 5.3 ACDC (Automated Cell-type Discovery and Classification through knowledge transfer)

ACDC is an algorithm primarily developed for the automated classification of canonical cell populations and the discovery of novel cell populations in CyTOF data [https://bitbucket.org/dudleylab/acdc (Lee *et al.*, 2017)]. Although it has been developed to be used on CyTOF data, it can also be useful in classifying cell populations in spatial IMC data due to the similarity of data composition between the two methods. It combines cell profile matching and semi-supervised learning to automate cell classification and discovery. By using *a priori* biological knowledge of canonical marker expression of known cell populations, ACDC offers reliable classification of known and discovery of new cell types in a semi-supervised manner (Lee *et al.*, 2017).

### 5.4 CELESTA (CELl typE identification with SpaTiAl information)

CELESTA is a machine learning cell classification and R-based pipeline [https://github.com/plevritis-lab/CELESTA (Zhang *et al.*, 2022]) designed primarily for the analysis of high multiplexed immunofluorescence data derived from CODEX (CO-Detection by indEXing) platform (Goltsev *et al.*, 2018). It is based on the Markov Random Field modeling and heavily relies on an operator input in the form of pre-defined knowledge of marker expression patterns of distinct cell classes (Zhang *et al.*, 2022). The whole concept is based on the assumption that cells in the tissues are organized in coherent spatial arrangements and that the cell neighborhoods carry essential information about cell identities residing inside the observed neighborhood. CELESTA, therefore, bases the cell classification on the marker expression patterns but also on the tissue architecture and spatial orientation of so-called ‘anchor cells’ (Zhang *et al.*, 2022). Anchor cells are cells that can be unquestionably defined and classified solely on their marker expression patterns that absolutely match prior biological knowledge. The rest of the cells that cannot be classified solely on their marker expression due to the ambiguity (referred to as ‘index cells’) are classified based on their position in regard to the anchor cells and their marker expression profiles (Zhang *et al.*, 2022).

The strength of this approach is that it does not rely solely on marker expressions to define cell identities, which in many cases can be ambiguous and unclear and can result in unprecise and flawed cell phenotyping. This method exploits spatial information to extrapolate information about cells whose identities are not clear from their marker expressions, and which might be mismatched by using other phenotyping approaches which are relying solely on cell marker expression.

### 5.5 CellSighter

CellSighter is a CNN-based tool for cell classification in high multiplex imaging data (Fig. 3) [https://github.com/KerenLab/CellSighter (Amitay *et al.*, 2022)]. It uses as input cell masks, multichannel .tiff files and a relatively small set of training images labeled by an expert to generate a ground-truth probability for each cell belonging to a specific cell class. The designers of CellSighter claim that this tool has an accuracy between 80% and 100% for the main cell types and also provides confidence in prediction as an output, allowing evaluation of cell classification quality (Amitay *et al.*, 2022). CellSighter can be successfully trained on a relatively small number of labeled examples and has been shown to reduce data overfitting (Amitay *et al.*, 2022). Although it is a powerful and useful tool for high-precision cell classification, that significantly reduces time spent on this task, it still demands prior expert knowledge and expert-guided cell class annotations of the train data set. It requires proficiency in using python and it does not take into consideration the tissue architecture as is the case with CELESTA.

### 5.6 Squidpy (Spatial Quantification of Molecular Data in Python)

Squidpy is a python algorithm built on the single-cell analysis framework Scanpy and anndata (Wolf *et al.*, 2018). It has been designed to aid spatial omics data analysis and visualization (Fig. 3) (Palla *et al.*, 2022). Squidpy combines a robust molecular data analysis framework with image analysis, providing a powerful and highly modular environment that can be easily joined with additional python data science and single-cell analysis tools for IMC data processing (Palla *et al.*, 2022). Although it has a capability for cell segmentation based on the watershed approach and integrated StarDist [(Schmidt *et al.*, 2018) https://github.com/stardist/stardist] and Cellpose [(Stringer *et al.*, 2021) https://github.com/mouseland/cellpose] algorithms (Palla *et al.*, 2022), it operates with anndata object output from the Steinbock and therefore can easily be implemented as a tool for downstream IMC data analysis after Steinbock cell segmentation (Windhager *et al.*, 2021). Squidpy offers extensive options for spatial analysis such as diverse methods in building the spatial graphs (e.g. based on KNN and radius expansion, or based on Delaunay triangulation), which are then used further for calculating co-occurrence across spatial dimensions (answers the question at which distances are distinct cell classes more likely to co-occur), interaction matrix (counts the edges that each cell shares with the others), neighborhood enrichment [answers the question which cell classes are more likely to be found in close proximity (enrichment) or to be further apart (avoidance)] and network centralities (answers the question on which cells are more often close with each other observed in the same group composition). Squidpy utilizes differential proximity testing Ripley’s statistics (L, F and G function) for assessing if, at a certain scale, cells have random, dispersed or clustered distribution and helps in determining the significance of occurring patterns (Palla *et al.*, 2022).

### 5.7 SIMPLI (Single-cell Identification from MultiPLexed Images)

SIMPLI is a Nextflow-based, platform-agnostic pipeline designed for the analysis of high-multiplexed spatial data (Bortolomeazzi *et al.*, 2022). It performs analysis of spatial data in three main steps. Each of these steps can be performed independently or skipped, giving an operator liberty to use input data from different sources in each specific analysis phase. The first step is raw image processing. In this phase, SIMPLI reads and processes raw data, preparing it for the second step in the process. The second step is the cell-based analysis which is divided into cell data extraction, cell phenotyping and spatial analysis. Cell data extraction is initiated with cell segmentation, based on CellProfiler or StarDist. Cell phenotyping can be performed using unsupervised approach, where unsupervised cell clustering is performed using Seurat (Bortolomeazzi *et al.*, 2022; Butler *et al.*, 2018) based on the marker expression values. The second approach for cell phenotyping is supervised, and it is based on user-defined thresholds of pixel intensities that are combined by logical operators for the identification of indicated cell phenotypes. Defined cell classes are then subjected to spatial analysis. In the third step (pixel-based analysis), areas positive for user-defined marker combinations are being quantified using the EBImage R package (Bortolomeazzi *et al.*, 2022; Pau *et al.*, 2010).

### 5.8 Graphical user interface software for IMC spatial data analysis

#### 5.8.1 HistoCAT (Histology Topography Cytometry Analysis Toolbox)

HistoCAT is a user-friendly and interactive downstream analysis tool developed by the Bodenmiller group for high multiplexed data analysis and visualization (Fig. 3) (Schapiro *et al.*, 2017). It has been built on the MATLAB environment, and it blends a high dimensional data visualization approach, methods for cell phenotyping and algorithms for intercellular interaction and neighborhood analysis (Schapiro *et al.*, 2017). The user interface is divided into two sections, a section for image visualization and a section for cytometry analysis.

The cytometry analysis section allows marker quantification and exploration of spatial single-cell data across the images, which can then be represented using a multidimensional reduction approach (t-SNE, PCA), histograms, scatter plots, heatmaps, etc. Detection of cell phenotypes can be assessed using two distinct approaches in histoCAT. The first approach is operator supervised and based on generating t-SNE maps, highlighting and manually gating the individual lineage markers on generated t-SNEs and by this, discriminating between distinct cell phenotypes (Schapiro *et al.*, 2017). The second approach is unsupervised and based on an unbiased PhenoGraph clustering algorithm (Levine *et al.*, 2015), where the intensity of each of the markers associated with each of the detected cell objects are being assessed, and cell phenotypes are automatically defined (Schapiro *et al.*, 2017).

Using spatial information for each of the identified cell phenotypes, histoCAT implements a dual approach for the exploration of significant intercellular interactions and specific cell neighborhoods. The first approach is operator guided and based on the assessment of cell phenotypes being in close proximity or in direct contact, and the second is based on the permutation (re-randomization) test (Schapiro *et al.*, 2017).

#### 5.8.2 ImaCytE

ImaCytE is a MATLAB-based, interactive software designed for IMC data analysis [https://github.com/biovault/ImaCytE (Somarakis *et al.*, 2021)]. This user-friendly tool helps in performing three distinct tasks.

1. IMC data quality control
  With this task, Imacyte assists the operator in identifying and excluding samples that are showing unreliable and unspecific staining patterns. This staining quality control is focused on discovering possible batch effects reflected by offset in the expression patterns and intensities of each marker used (Somarakis *et al.*, 2021).
2. Cell phenotype identification
  Imacyte utilizes the t-SNE approach for dimensionality reduction (Linderman and Steinerberger, 2019; Somarakis *et al.*, 2021). ImaCytE represents and arranges clusters in the heatmap hierarchically, based on their similarity in the median expression of each marker used, and provides a specific two-level color-coding for better visualization of each cluster and its subclusters (based on the marker expression similarities) (Somarakis *et al.*, 2021). This helps in verification of the biological significance of given clusters by representing them as tissue structures in the image view as colored cell objects.
3. Cell microenvironment exploration
  This task is divided into two steps. The first step is spatial interactions overview, where an operator can get a global scale overview of intercellular interactions and get an idea of which phenotypes are more frequently interacting with each other. The second step is spatial interaction details, where an operator has the possibility to undertake a detailed exploration of specific cell niches and determine their biological significance and frequency of occurrence (Somarakis *et al.*, 2021).

The main strength of using ImaCytE for IMC data analysis lies in the implemented quality control function, which allows quick and easy exclusion of flawed samples and markers. ImaCytE has implemented a powerful tool for niche exploration and intuitive representation of the niches in the form of donut-like glyphs, giving a robust visual overview of niche composition.

#### 5.8.3 SPEX (Spatial Expression Explorer)

SPEX is a comprehensive, end-to-end graphical user interface (GUI) tool designed for the analysis of spatial omics data (Pechuan-Jorge *et al.*, 2022). Its flexible, modular landscape and GUI interface allow simple implementation and blending of various user-defined analysis algorithms, preferred based on the project's needs. As of now, this tool is able to perform a wide range of tasks in image analysis, such as cell segmentation, cell classification (phenotyping), analysis of spatial marker expression patterns and exploration of intercellular spatial interactions (Pechuan-Jorge *et al.*, 2022).

Image processing in SPEX consists of five distinct steps. These steps are loading the data, preprocessing images, cell segmentation, post-processing and feature extraction (Pechuan-Jorge *et al.*, 2022). Each of these steps is modular, meaning that an operator can decide which submodules to be used to address each of the tasks in the best possible way depending on the type of data and what’s suitable for further analysis. The image preprocessing step consists of multiple submodules one can choose from, such as non-local mean (NLM) deionizing, background subtraction or median blur filter submodule to denoise images and enhance pixel data (Pechuan-Jorge *et al.*, 2022). Cell segmentation in SPEX can be performed by choosing between different submodules such as watershed algorithm, Cellpose, StarDist or Mesmer (Pechuan-Jorge *et al.*, 2022). The post-processing module has a task to help correct for artifacts in cell segmentation and modify the single-cell segmentation labels (Pechuan-Jorge *et al.*, 2022). Feature extraction module allows an operator to extract single-cell features such as mean intensity for each channel determined per each cell. This gives a cell by expression matrix output which is used as an input for downstream steps (clustering module) (Pechuan-Jorge *et al.*, 2022). The clustering module allows the user to cluster identified cells per sample (with cluster alignment between samples) or across all the samples (bulk clustering). Cell phenotype labels are then defined by examining both the marker expression patterns and spatial orientation of each defined cluster. After defining cell populations, SPEX uses a spatial analysis module to explore spatial intercellular interactions (Pechuan-Jorge *et al.*, 2022).

As it implements all the necessary analysis steps, gives freedom to choose between multiple analysis pipelines and has an intuitive and user-friendly interface, SPEX represents an excellent, all-encompassing tool for dealing with IMC data, capable of performing rather complex analysis in a simple and time-efficient manner.

#### 5.8.4 CytoMAP

CytoMAP is a MATLAB-based tool designed for the analysis of high multiplex data (Stoltzfus *et al.*, 2020). It combines already available analytical tools for single-cell data clustering, dimensionality reduction, positional correlation and identification of localized cellular networks and microniches, into a user-friendly GUI-based software (Stoltzfus *et al.*, 2020). Compared to ImaCytE, CytoMAP allows a higher degree of freedom when interacting with the data and more possibilities for spatial analysis. Although CyToMAP is GUI-based and user-friendly, ImaCytE might still be simpler, better organized and easier to use for beginners (Fig. 3).

## 6 Concluding remarks

The analysis of IMC data can be roughly divided into four phases: data visualization and conversion, data preprocessing, cell segmentation and downstream analysis (Fig. 3). Here, we need to remember and keep in mind that data obtained using the IMC platform are not classical micrographs. Data obtained by IMC are numerical and represent the number of ion counts detected in two-dimensional space. Different approaches can be used to transform this type of data into pseudo-micrographs that can be subsequently subjected to micrograph-like image analysis techniques. Among the available tools discussed in this review, the author strongly prefers MCD viewer for raw data visualization and data quality assessment, and readimc python package for conversion of MCD raw data to ome.tiff data format.

Data preprocessing is an important step in IMC image analysis. The best way to handle channel crosstalk is to use the Bodemillers’ CATALYST pipeline before each data acquisition. Another highly recommended approach to correct for artifacts is IMC-denoise (Fig. 3).

Cell segmentation is the most critical step in image analysis. Some of the most advanced techniques, promising to give the most accurate cell delineations, are tools based on deep learning algorithms. Curated and trained on large amounts of data and constantly retrained by an increasing number of everyday users, these tools guarantee the best possible segmentations capturing the complex morphology of the cells. How successful each of the available cell segmentation pipelines will be, largely depends on the data and quality of the staining used for cell segmentation. The author finds the Steinbock cell segmentation pipeline utilizing the Mesmer algorithm as a quick, easy and powerful cell segmentation tool which, when used in combination with IMC Cell Segmentation Kit (Standard BioTools, South San Francisco, USA), gives very precise cell masks (Fig. 3). Whichever cell segmentation approaches are to be used, it is strongly advisable to quality check the cell outlines by overlaying the cell masks over the original images.

Cell phenotyping is another critical step in assuring the correctness and validity of the output image analysis data. The introduction of spatial information, as is the case with e.g. CELESTA, can indeed help increase the precision of cell phenotyping, helping in the correct resolution of the cell identities by applying spatial information. Some available tools offer a semi-supervised approach for cell classification, such as e.g. histoCAT, which offers visually guided manual gating of the cells on the .tiff image, or on the UMAP/t-SNE scatterplot, where an experienced operator can follow the spatial position of the gated cells and compare the patterns with *a priori* knowledge.

As IMC gives a robust spatial resolution of the data, it creates an opportunity to be exploited for comprehensive spatial analysis. For example, if one would like to answer a question about which specific types of cells are more likely to co-occur together, or which cell types are likely to avoid each other, several available tools give a possibility to study attraction and avoidance between individual cell phenotypes. This is being performed by detecting direct cell–cell interactions, or by detecting a certain cell phenotype that might be residing in a defined perimeter from the central cell phenotype. Useful tools to accomplish these tasks would be quidpy, histoCAT or ImaCytE. On the other side, if one would like to answer the question of how groups of cells interact and if defined specific interacting cell groups (cell neighborhoods) are associated with certain pathological states, imcRtools, spicyR or lisaClust would be the suggested approach.

For beginners, the author would single out ImaCytE as one of the excellent tools, which seems that, at this point, by its comprehensiveness, simplicity, possibility to directly overview and explore various cell–cell interactions and implemented niche exploration, surpasses other available beginner friendly tools. Nevertheless, squidpy, lisaClust, SpicyR and imcRtools offer more comprehensive spatial profiling, but they require at least basic proficiency in using python and R coding.

At this point, different phases of image analysis require utilization and transfer between different analysis platforms (Fig. 3). As IMC is gaining in popularity, there will be an increasing number of tools developed with attempt to give a more compact unified solution to IMC data analysis. Some new, already available tools indeed promise modularized all-in-one analysis platforms that could perform tasks from image pre-processing and cell segmentation to spatial data analysis using interoperability between specific task modules, as an example here already described SPEX. Although, as different tools dedicated to different phases of IMC analysis are being frequently developed and improved, it can be challenging to create a robust all-in-one, a standalone tool, able to perform all the necessary tasks and in the same time to be easily improvable, so it can go in step with the increased development of novel and improved software packages. Visiopharm (Hørsholm, Denmark), in cooperation with Standard BioTools (South San Francisco, USA) is already making efforts in developing a commercial, standalone, GUI tool for IMC data analysis. Although having in mind the aforementioned difficulties, it could be challenging to surpass the freeware projects that allow shifting between and combining different tools, enabling freedom in data analysis and easy implementation of improved solutions. All the tools described here are interoperable and provide freedom in combining different software solutions for each step of the analysis. Frameworks such as csverse used for python tools and Bioconductor used for R tools, support increased interoperability between a variety of present and newly developed tools.

When choosing which tool to use for data analysis, another important thing to consider is the amount of data that needs to be processed. Some of the described tools do have problems when a big number of images are planned to be processed. Generally, MATLAB-based tools (e.g. HistoCAT and ImaCytE) suffer from scalability issues and might encounter difficulties when used on big datasets. Some approaches, e.g. IMC-Denoise require very strong hardware settings to be even able to run. On the other side, web-based tools, python-based and R-based tools are usually capable of handling big data, and although HistoCAT and ImaCytE are very user-friendly and easy to use by a beginner with little or no experience in bioinformatics, later should be prioritized for big datasets.

Key points

- Imaging Mass Cytometry (IMC) is a strong asset for in depth study of histology, pathophysiology and tumor microenvironment.
- IMC-derived data are not classical micrographic images and therefore they require slightly different approach in analysis.
- IMC data analysis is a complex process consisting of multiple phases such as data visualization and transformation, data pre-processing, cell segmentation and downstream analysis.
- As the number of studies based on IMC increase, so is the number of available tools and pipelines designated solely for IMC data analysis. The author with this review wanted to provide a comprehensive compendium on tools and pipelines available and provide a guidance to researchers stepping in this newly emerging field.
